# Metabolic reprogramming and immune microenvironment characteristics in laryngeal carcinoma: advances in immunotherapy

**DOI:** 10.3389/fimmu.2025.1589243

**Published:** 2025-04-30

**Authors:** Kexin Ma, Qingjie Mao, Bing Fei, Tingting Ni, Zhenxin Zhang, Haosheng Ni

**Affiliations:** ^1^ Affiliated Hospital of Nantong University, Medical School of Nantong University, Nantong, China; ^2^ Department of Otolaryngology, Affiliated Hospital of Nantong University, Medical School of Nantong University, Nantong, China; ^3^ Department of Otolaryngology Head and Neck Surgery, Affiliated Huai’an Hospital of Xuzhou Medical University, Huai’an, China; ^4^ Department of Oncology, Nantong Tumor Hospital, Nantong, China

**Keywords:** laryngeal squamous cell carcinoma, metabolic reprogramming, tumor microenvironment, immune checkpoint inhibitors, glycolysis, precision medicine, immunotherapy

## Abstract

Laryngeal squamous cell carcinoma (LSCC) is a prevalent malignancy with high mortality and recurrence rates, necessitating novel therapeutic strategies. Recent research highlights the pivotal role of metabolic reprogramming and immune microenvironment alterations in LSCC pathogenesis, providing promising avenues for targeted therapy. This review summarizes the metabolic characteristics of LSCC, including glycolysis, lipid metabolism, and amino acid biosynthesis, and their implications for tumor progression and therapeutic resistance. Additionally, this review further describes the tumor microenvironment’s immunosuppressive landscape, including immune checkpoint regulation, tumor-associated macrophages, and T-cell dysfunction. The integration of metabolic and immune-targeted strategies represents a promising frontier in LSCC treatment, warranting further investigation.

## 1 Introduction

Laryngeal squamous cell carcinoma (LSCC) is a prevalent and aggressive malignancy, representing approximately one-third of head and neck cancers (1, 2). Recent research highlights the pivotal roles of metabolic reprogramming and the tumor immune microenvironment (TME) in cancer progression (3–6). Metabolic reprogramming, involving alterations in glucose, lipid, and amino acid metabolism, supports tumor growth, survival, and therapy resistance (7–12). Simultaneously, the TME, comprising immune cells, stromal components, and extracellular matrix elements, facilitates immune evasion and tumor progression (13–15). Immunotherapy, especially immune checkpoint inhibitors (ICIs) like pembrolizumab and nivolumab, has transformed the treatment of recurrent or metastatic LSCC, improving survival and quality of life (16). However, variability in treatment response and resistance mechanisms necessitate a deeper understanding of the metabolic and immune landscape of LSCC to optimize therapeutic outcomes.

This review summarizes the metabolic characteristics of LSCC, including glycolysis, lipid metabolism, and amino acid biosynthesis, and their implications for tumor progression. We also examine the immunosuppressive features of the TME, such as immune checkpoint regulation and T-cell dysfunction, and their impact on immunotherapy. Finally, we provide the advances in immunotherapy and the potential of integrating metabolic and immune-targeted strategies to enhance precision medicine in LSCC management. By synthesizing current knowledge, this review aims to guide the development of more effective treatments for LSCC.

## 2 The tumor microenvironment in laryngeal squamous cell carcinoma

### 2.1 Composition of the tumor microenvironment in laryngeal squamous cell carcinoma

The tumor microenvironment is a complex ecosystem shaped by interactions among malignant cells, cancer stem cells (CSCs), and stromal components, including vascular-associated cells and extracellular matrix (ECM) elements, during tumorigenesis and progression (17–20). This environment undergoes metabolic reprogramming, influencing gene expression, cellular differentiation, and tumor cell functionality. CSCs, a rare but critical subpopulation with self-renewal capacity (3, 7), play a key role in tumor recurrence and metastasis. The stromal compartment of the TME includes non-immune and immune cells. Non-immune stromal cells, such as fibroblasts, endothelial cells, and pericytes, provide structural and metabolic support. Immune cells, including lymphocytes and macrophages, facilitate immune evasion and promote immune tolerance (13). Cancer-associated fibroblasts (CAFs) are particularly significant due to their role in ECM remodeling and supporting LSCC proliferation. Key immune subsets, such as dendritic cells, tumor-infiltrating lymphocytes (TILs), and tumor-associated macrophages (TAMs), exert diverse immunomodulatory effects (16).

Non-cellular TME components include ECM proteins, cytokines, chemokines, growth factors, proteases, and non-coding RNAs (21–29). ECM proteins regulate biomechanical properties, influencing cancer cell adhesion, survival, differentiation, and invasion. Secreted factors create a pro-angiogenic and immunosuppressive landscape, promoting tumor progression. Non-coding RNAs, implicated in LSCC radio-resistance (30), show potential as diagnostic and prognostic biomarkers, with their downregulation linked to reduced tumor proliferation and metastasis.

### 2.2 Tumor microenvironment and the development of laryngeal squamous cell carcinoma

Cancer progression involves genetic alterations (31, 32), such as oncogene overexpression and tumor suppressor gene silencing, leading to epithelial cell changes and precancerous lesions (33–35). TME promotes tissue invasion, metastasis, and immune evasion, facilitating malignant transformation (36–40). Recent studies highlight key TME components in laryngeal squamous cell carcinoma (LSCC) progression, identifying potential therapeutic targets (**Table 1**). Genomic analyses link LSCC risk to overexpression of SRY-box transcription factor 2 (SOX2), cortactin (CTTN), and focal adhesion kinase (FAK) (41, 42). SU (43) identified CD163+ TAMs and Ki-67 proliferation as dysplasia severity indicators, with Ki-67 facilitating spheroid formation, offering new predictors for LSCC risk stratification. LSCC invasion and metastasis involve complex mechanisms. KLOBUCAR (44) linked ladinin-1 to MIF-CD44-β1 integrin signaling, increasing LSCC cell motility. TOPF (45) demonstrated LSCC-derived factors in lymph nodes promote CD163+ TAMs, raising nodal metastasis risk. Immune evasion is critical in LSCC. Elevated Tregs in LSCC patients suppress CD4+ and CD25+ T cell proliferation (46). WEN (47) found IL-33 increases Foxp3+ GATA3+ Tregs and suppresses T cell proliferation via IL-10 and TGF-β1, with ST2 inhibition reversing this effect. PD-L1 overexpression in LSCC binds PD-1, inducing T cell anergy or apoptosis. Yu et al. (48) noted higher PD-L1 levels in LSCC, negatively correlating with CD8+ TILs and CD16+ M1 TAMs but positively with CD206+ M2 TAMs.

**Table 1:** Key components of the LSCC tumor microenvironment and their functional roles.

Component	Subtype/Category	Functional Role in LSCC	Clinical/Therapeutic Implications
Immune Cells	CD8+ T cells	Primary antitumor effector cells; often functionally exhausted in TIME	Target for checkpoint inhibitors
	Tregs (Foxp3+)	Immunosuppression via IL-10/TGF-β; correlate with advanced stage	ST2/IL-33 axis inhibition potential
	M2 TAMs (CD163+/CD206+)	Promote angiogenesis, metastasis; associate with nodal involvement	CSF1R inhibitors in clinical trials
Stromal Cells	Cancer-associated fibroblasts	ECM remodeling, cytokine secretion (TGF-β, HGF); drive treatment resistance	FAK targeting strategies
	Endothelial cells	Angiogenesis (VEGF-dependent); lymphatic spread facilitation	VEGF inhibitors (e.g., bevacizumab)
Soluble Factors	PD-L1	Immune checkpoint ligand; induces T cell anergy	Predictive biomarker for anti-PD-1 response
	Lactate	Metabolic byproduct; promotes M2 polarization and T cell dysfunction	MCT4 inhibitors in development
ECM Components	Collagen fibers	Physical barrier; mechanosignaling via integrins	LOX family inhibitors
	Fibronectin	Metastatic niche formation	α5β1 integrin antagonists

These findings illustrate the multifaceted mechanisms by which LSCC invades surrounding tissues and metastasizes. Building upon this mechanistic understanding, recent studies have identified biomarkers that may assist in risk stratification and prognosis of LSCC. Prognostic biomarkers in LSCC include CD3, CD8, CD57, and S100, correlating with better outcomes, while CTLA-4 predicts poor prognosis (49). Tumor-stroma interactions, including stroma-rich tumors and fibroblastic patterns, indicate aggressive disease (50). Inflammatory factors regulate disease progression and significantly influence the efficacy of therapies (51–56). Vassil et al. (57) found PD-L1 expression correlates with TIL density and survival, which was also observed in IL-12Rβ2+ TILs (58). Further, Schulter et al. (59) identified CD31-positive vasculature and VEGF as markers for recurrence risk.

## 3 Metabolic characteristics of the microenvironment in laryngeal squamous cell carcinoma

The tumor microenvironment (TME) in cancer undergoes dynamic alterations in cellular and extracellular components, driving metabolic reprogramming—a hallmark of malignancy (60–63). Metabolomics, utilizing mass spectrometry (MS) or nuclear magnetic resonance (NMR) spectroscopy, enables comprehensive profiling of metabolites in biological specimens, aiding in biomarker identification and elucidating disease mechanisms (64). NMR was used to analyze LSCC tissues, revealing elevated lactate, amino acids, choline compounds, creatine, taurine, and glutathione, alongside reduced triglycerides (64). Fei et al. (65) employed GC-TOF-MS and UPLC-TOF-MS to identify 41 differentially expressed metabolites in LSCC tissues, including upregulated TCA cycle intermediates, lactate, purine, and pyrimidine metabolites, and downregulated fatty acid derivatives. Urine metabolomics further distinguished LSCC patients from controls, highlighting pantothenic acid, palmitic acid, myristic acid, oleamide, sphingosine, and phytoglycine as potential diagnostic biomarkers (66). These findings underscore the role of lipid metabolism in bio-membrane biosynthesis and cancer cell proliferation. Current studies emphasize glycolysis, lipid metabolism, and amino acid biosynthesis as key metabolic features of LSCC, aligning with genomic and proteomic insights (67). These findings highlight the importance of metabolic reprogramming in LSCC pathogenesis and the potential of metabolomics for identifying therapeutic targets and biomarkers.

### 3.1 Glucose metabolism

The Warburg effect, as a hallmark of cancer metabolism, involves a preference for glycolysis over oxidative phosphorylation for ATP production, even under aerobic conditions (68). This metabolic shift promotes radiation resistance and malignant progression in cancers like LSCC. Glycolysis is initiated by glucose uptake via glucose transporter-1 (Glut-1) and phosphorylation by hexokinase-II (HK-II), with pyruvate converted to lactate by lactate-dehydrogenase (LDH) and exported via monocarboxylate transporter-4 (MCT-4) to prevent intracellular acidification (69). Glut-1, a key driver of the Warburg effect, was studied by WANG (70) in Hep-2 cells, revealing that aberrant WISP1 expression enhanced glucose uptake, lactate production, and cisplatin resistance by upregulating YAP1 and TEAD1. LU (71) demonstrated that CRISPR/Cas9-mediated knockout of HIF-1 and Glut-1 impaired glucose uptake and LSCC progression. HU (72) linked PCK2 downregulation to suppressed LSCC progression and Glut-1 interaction.

HK-II, regulated by lncRNA loc285194 (73), miR-125a (74), and miR-125b-5p (75), is central to glycolysis. XU (76) found that lncRNA PCAT19 suppresses PDK4 via the miR-182/PDK4 axis, promoting glycolysis. LIU (77) showed that FOXJ1 knockdown attenuates glycolysis by inhibiting the Wnt/β-catenin pathway. MCT-4 facilitates lactate efflux, while MCT-1 mediates uptake, creating metabolic coupling (78). CURRY (79) observed high MCT-4 and low MCT-1 expression in tumor cells, promoting glycolysis. Targeting MCT-4 offers therapeutic potential. WANG (69) linked Glut-1, MCT-4, and CAIX expression to LSCC grade, with combined inhibition suppressing glycolysis. LIU (67) identified RASSF1, PGK1, CAII, and CAXII as key metabolic regulators in LSCC.

### 3.2 Lipid metabolism

Lipid-mediated signaling pathways are crucial in LSCC pathogenesis. Cancer cells upregulate fatty acid and phospholipid biosynthesis, producing metabolites that modify membrane components and act as signaling molecules. Fatty acid biosynthesis begins with citrate export from mitochondria, converted to acetyl-CoA by ATP citrate lyase. Acetyl-CoA carboxylase (ACC) then generates malonyl-CoA, which fatty acid synthase (FAS) converts to palmitic acid, a precursor for other fatty acids. FAS (80) and ACC (81) are overexpressed in LSCC, highlighting their role in fatty acid biosynthesis and potential as prognostic markers. Fatty acid desaturase 1 (FADS1), a key enzyme in polyunsaturated fatty acid biosynthesis, converts linoleic acid to arachidonic acid (AA). Zhao (82) found elevated FADS1 in LSCC tissues, with knockdown impairing cell proliferation, migration, and invasion, suggesting FADS1 promotes LSCC via AKT/mTOR signaling.

Lipid metabolism plays a crucial role in laryngeal squamous cell carcinoma (LSCC) progression through its interplay with inflammation and oncogenic signaling pathways. Arachidonic acid (AA), released from membrane phospholipids by phospholipase A2 (PLA2), serves as a substrate for cyclooxygenase (COX) and lipoxygenase (LOX) to generate pro-inflammatory eicosanoids (83, 84). Studies have demonstrated elevated expression of PLCγ-2 and LOX-12 in LSCC, which correlates with advanced clinical stage, poor differentiation, and metastatic potential, while COX-2 overexpression has been associated with tumor recurrence (85). Early-stage LSCC exhibits increased levels of linoleic acid (LA), AA, and saturated fatty acids that enhance LOX and COX-2 activity, driving oxidative stress, inflammatory responses, angiogenesis, and immune evasion through upregulation of NF-κB and Bcl-2 (86, 87). Notably, reduced PTEN expression in LSCC tissues serves as a prognostic indicator, and DJ-1 silencing has been shown to restore PTEN expression, thereby inhibiting tumor cell proliferation and invasion (88–90). These findings collectively identify FAS, ACC, LOX-12, COX-2, and PTEN as critical regulators in LSCC lipid metabolism, highlighting the therapeutic potential of targeting fatty acid biosynthesis, AA metabolism, and the PTEN/PI3K/AKT/mTOR axis in LSCC treatment (86).

### 3.3 Nitrogen metabolism

Nitrogen is essential for proteins, DNA, and RNA. In humans, it is primarily used for urea biosynthesis in the liver, with disruptions in the urea cycle common in tumors, leading to upregulated pyrimidine biosynthesis and amino acid metabolism (91). The lysyl oxidase (LOX) family stabilizes collagen and elastic fibers, regulating EMT and tumor progression (92). Elevated LOX expression correlates with poor prognosis and metastasis in LSCC (93). Tryptophan metabolism via indoleamine 2,3-dioxygenase (IDO) produces kynurenine, an immunosuppressive metabolite. ENGIN (94) found higher IDO activity in advanced LSCC, with elevated serum neopterin post-resection indicating poor outcomes. Adenosine, an extracellular signaling molecule, activates tumor cell receptors, promoting growth. WILKAT (95) showed A2B receptor inhibition reduces tumor growth and vascularization. Hypoxia and inflammation in the TME increase adenosine production, enhancing immunosuppression. CD39 and CD73, elevated in head and neck cancer, accelerate ATP hydrolysis, increasing adenosine and reinforcing immunosuppression (96).

Moreover, metabolic reprogramming shapes the immune microenvironment by modulating nutrient availability, altering metabolite composition, and producing immunosuppressive byproducts. For instance, lactate accumulation due to enhanced glycolysis impairs CD8^+^ T cell and NK cell cytotoxicity while promoting regulatory T cell (Treg) differentiation. Lipid accumulation in the TME compromises dendritic cell function and fosters M2-like macrophage polarization, promoting immune evasion. Elevated IDO activity in tryptophan metabolism generates kynurenine, suppressing T cell proliferation and driving Treg expansion. These alterations jointly contribute to immune dysfunction and resistance to immune checkpoint inhibitors in LSCC (**Table 2**).

**Table 2:** Metabolic reprogramming In LSCC.

Metabolic reprogramming pathway	Regulatory targets	Change
Glycolysis	Glut-1, HK-II, PDK4, MCT-4, CAIX	↑
	WISP1/YAP1/TEAD1	↑
	HIF-1, Glut-1	↑
	PCK2	↓
Lipid Metabolism	FAS, ACC, FADS1	↑
	LOX-12, COX-2	↑
	PI3K/AKT/mTOR axis	↑
	PTEN	↓
Nitrogen Metabolism	LOX	↑
	IDO	↑
	CD39	↑
	CD73	↑

↓ means downregulated, ↑ means upregulated.

## 4 Advances in immunotherapy for laryngeal squamous cell carcinoma

### 4.1 Cytotoxic agent

Cytotoxic agents such as cisplatin, 5-fluorouracil (5-Fu), and docetaxel play a central role in laryngeal preservation strategies, exerting antineoplastic effects through mechanisms involving DNA damage or inhibition of protein synthesis (97). Cisplatin, a cornerstone in the treatment of LSCC, demonstrates substantial efficacy but is limited by dose-dependent nephrotoxicity. Similarly, 5-Fu, a widely utilized chemotherapeutic for solid tumors, carries a cardiotoxicity risk ranging from 0% to 35% (98). Docetaxel, effective against various metastatic malignancies, is frequently associated with peripheral neuropathy, alopecia, and neutropenia—adverse effects that often necessitate dose modifications (99). Clinical studies have provided supportive evidence for laryngeal preservation. Urba et al. Urba et al. (100) reported a 3-year laryngeal preservation rate of 70% using induction chemotherapy, exceeding the 64% rate observed in the Veterans Affairs trial (101). The RTOG 91–11 trial and its follow-up corroborated these findings (102, 103). Notably, incorporation of docetaxel into the cisplatin and 5-Fu induction regimen—forming the TPF regimen—has demonstrated superior outcomes. The TPF regimen yielded improved laryngeal preservation rates at both 5 years (74% vs. 58%) and 10 years (70% vs. 47%) compared to PF alone (104), establishing TPF as a more effective induction strategy (105, 106).

However, concerns regarding patient selection and generalizability have emerged. Olsen (107) emphasized that participants in these trials were generally younger and presented with limited nodal disease, potentially limiting the applicability of findings to broader patient populations. Furthermore, conflicting evidence has challenged the universal adoption of laryngeal preservation protocols. For instance, Nocon (108) and Bates (109) reported improved survival with total laryngectomy. Dyckhoff (110) further noted that patients with T4-stage LSCC treated with chemoradiotherapy exhibited a twofold increase in mortality risk compared to those receiving total laryngectomy followed by adjuvant radiotherapy. Collectively, these findings underscore the importance of individualized treatment planning, as the heterogeneity in tumor staging, patient characteristics, and treatment response continues to constrain the universal implementation of induction chemotherapy or concurrent chemoradiotherapy as a standard approach.

### 4.2 Epidermal growth factor receptor monoclonal antibodies

Monoclonal antibodies targeting the EGFR, such as cetuximab and nimotuzumab, are the only clinically approved targeted therapies for head and HNSCC (111). EGFR is overexpressed in approximately 90% of HNSCC cases, and its aberrant activation in laryngeal carcinoma promotes uncontrolled proliferation, radiotherapy resistance, and poor prognosis (112, 113). Physiologically, EGFR regulates epidermal cell development, with expression limited to undifferentiated basal keratinocytes and diminishing as cells migrate to the epithelial surface. Activation by ligands like epidermal growth factor (EGF) and TGF-α triggers tyrosine kinase signaling, driving growth-associated transcription in normal and malignant cells. Cetuximab and nimotuzumab competitively inhibit EGFR activation, disrupting downstream signaling, reducing cellular survival, and enhancing tumor-targeting efficacy compared to conventional chemotherapy.

EGFR inhibition enhances radiosensitivity in laryngeal carcinoma, improving radiotherapy outcomes (114). Cetuximab combined with radiotherapy extended locoregional control to 24.4 months versus 14.9 months with radiotherapy alone, likely due to enhanced apoptosis without increased toxicity (115). Bonner et al. (116) reported a 3-year laryngeal preservation rate of 87.9% with cetuximab plus radiotherapy, compared to 76.8% with radiotherapy alone. Similarly, Noronha et al. (117) observed a 74.1% laryngeal preservation rate with nimotuzumab combined with cisplatin and 5-fluorouracil (5-FU), supporting anti-EGFR antibodies’ role in organ preservation (118). However, the RTOG 10–16 trial found lower 5-year overall survival with cetuximab plus radiotherapy (77.9%) versus cisplatin plus radiotherapy (84.6%) in HPV-positive laryngeal carcinoma (119). While cetuximab is less effective than cisplatin in cisplatin-tolerant patients, it remains a viable alternative for cisplatin-resistant or intolerant individuals. Despite its clinical utility, cetuximab benefits only a subset of patients, underscoring the importance of patient selection in optimizing EGFR-targeted therapy.

### 4.3 Immune checkpoint inhibitors

Immune checkpoint inhibitors have significantly advanced the treatment of cancer (120–123), particularly for recurrent or metastatic cases. In 2016, the FDA approved PD-1 inhibitors nivolumab and pembrolizumab for platinum-refractory recurrent/metastatic HNSCC (124). These inhibitors counteract tumor immune evasion by restoring T-cell-mediated cytotoxicity, targeting the PD-1/PD-L1 axis, a key regulator of T-cell activation. PD-1, expressed on immune cells, interacts with PD-L1 to suppress T-cell activity via the PI3K-AKT pathway, promoting immune tolerance (125, 126). Nivolumab and pembrolizumab block PD-1, preventing immune suppression and enhancing antitumor immunity.

Pembrolizumab’s efficacy was first shown in the KEYNOTE-012 trial (127), with the phase III KEYNOTE-048 trial (128) confirming its role as a first-line treatment. Recently, a randomized, double-blind, phase 3 trial evaluated the efficacy and safety of the PD-1 monoclonal antibody finotonlimab (SCT-I10A) combined with cisplatin plus 5-fluorouracil (C5F) as first-line treatment for recurrent HNSCC (NCT04146402). In the finotonlimab plus C5F group, the median OS was 14.1 months, compared with 10.5 months in the placebo plus C5F group. This study highlights the effectiveness of immunotherapy combined with chemotherapy in recurrent or metastatic head and HNSCC (129). Furthermore, clinical studies have shown that PD-L1-high HNSCC patients treated with a PD-L1 inhibitor combined with 5-azacytidine (5-aza) experienced a significant extension in overall survival (OS) (NCT03019003) (130). To date, numerous clinical trials are still underway, evaluating the efficacy and optimal dosage of immune checkpoint inhibitors, exploring other immune targets, and providing new therapeutic targets for the treatment of laryngeal cancer. In addition to PD-1, other immune checkpoints such as cytotoxic T-lymphocyte-associated protein 4 (CTLA-4) and lymphocyte activation gene-3 (LAG-3) are being investigated (131, 132).

## 5 Conclusion

Laryngeal squamous cell carcinoma (LSCC) is characterized by profound metabolic reprogramming and an immunosuppressive tumor microenvironment, both of which contribute to disease progression and therapeutic resistance. Immunotherapy, particularly immune checkpoint inhibitors (ICIs), has demonstrated substantial promise in treating recurrent or metastatic LSCC. However, limitations in response rates and the development of resistance necessitate combinatorial strategies.

Recent preclinical and early-phase clinical studies have highlighted the potential of combining metabolic inhibitors with ICIs to enhance antitumor immunity. For instance, co-administration of PD-1 inhibitors with glycolytic inhibitors such as 2-deoxy-D-glucose (2-DG) has been shown to restore CD8^+^ T cell function and reduce tumor burden in murine models of head and HNSCC, including LSCC. Similarly, targeting lipid metabolism using fatty acid oxidation (FAO) inhibitors like etomoxir in combination with PD-1 blockade has led to augmented T cell infiltration and enhanced antitumor efficacy in preclinical studies. In clinical contexts, a phase I trial combining pembrolizumab with the glutaminase inhibitor CB-839 (telaglenastat) in solid tumors demonstrated favorable safety and preliminary antitumor activity, supporting the translational relevance of metabolic-immune co-targeting strategies. These findings underscore the therapeutic potential of dual modulation of tumor metabolism and immune checkpoints, offering a promising avenue for overcoming immune resistance and improving clinical outcomes in LSCC. Future research should aim to delineate optimal combinations, identify predictive biomarkers, and validate efficacy through large-scale clinical trials. Integrating metabolic and immune-targeted therapies represents a rational and potentially transformative approach for precision medicine in LSCC treatment.
